# Myocardial function and perfusion assessment with exercise stress cardiovascular magnetic resonance using an MRI-compatible treadmill in patients referred for stress SPECT

**DOI:** 10.1186/1532-429X-14-S1-P1

**Published:** 2012-02-01

**Authors:** Paaladinesh Thavendiranathan, Jennifer Dickerson, Debbie Scandling, Vijay Balasubramanian, Nathan Hall, Eric Foster, John W Arnold, Michael Pennell, Orlando P Simonetti, Subha V Raman

**Affiliations:** 1 Cardiovascular Medicine, Cleveland Clinic Foundation, Cleveland, OH, USA; 2 Cardiovascular Medicine, The Ohio State University, Columbus, OH, USA

## Background

Exercise stress cardiac magnetic resonance (CMR) has recently become feasible with the development of a fully MRI-compatible treadmill system along with improvements in imaging techniques. The utility of this setup has not been systematically compared with nuclear perfusion imaging. The study objective was to evaluate the accuracy and prognostic value of exercise stress CMR with a treadmill placed immediately next to the MRI scanner table in patients referred for treadmill stress nuclear perfusion imaging.

## Methods

**Figure 1:**
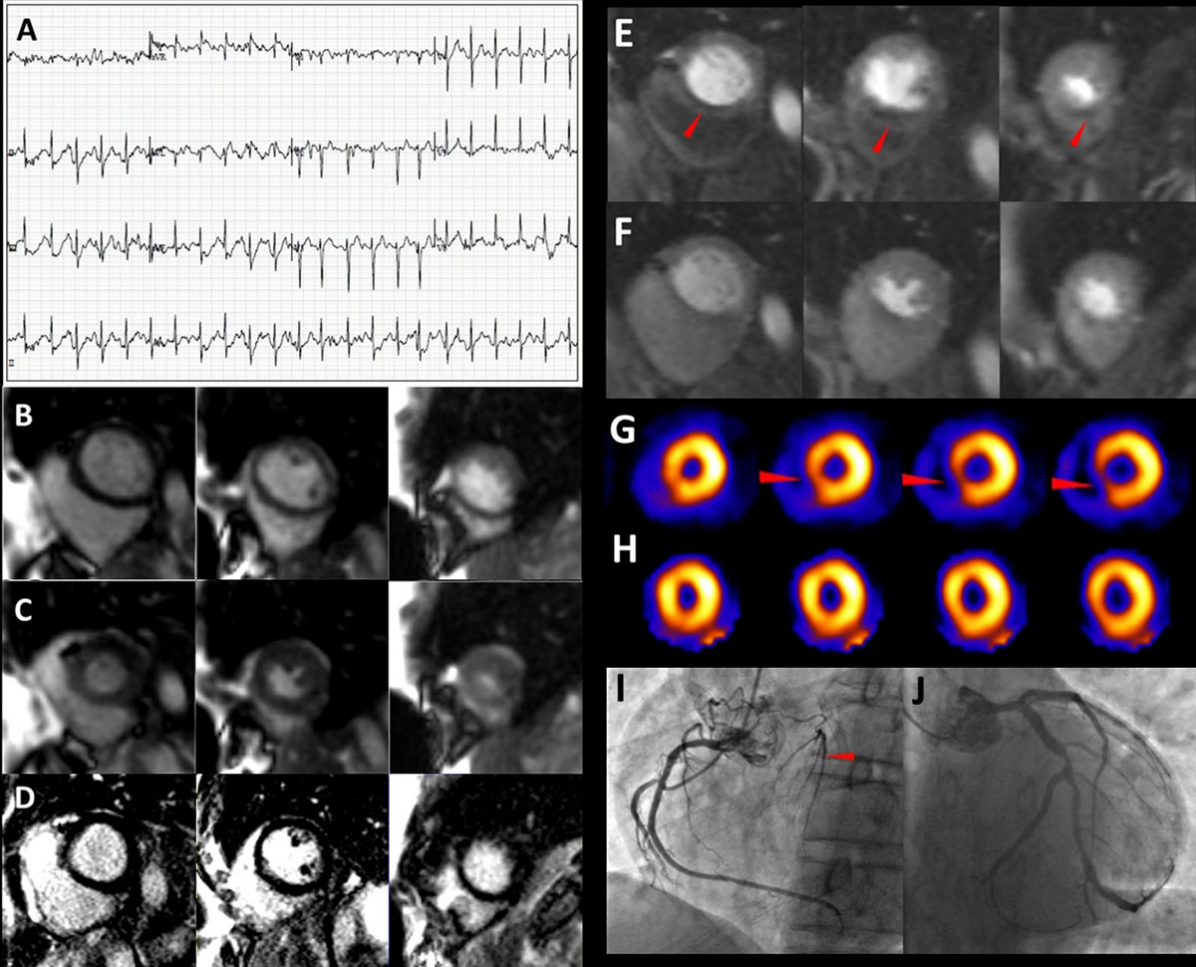
Ischemia by treadmill stress CMR in a 59-year-old male with known history of CAD with new onset chest pain. At peak exercise, no ST changes diagnostic of ischemia were present (panel A). No wall motion abnormalities were present on peak stress cines (panel B, end diastolic frame; panel C, end systolic frames in three slice positions). Delayed enhancement imaging did not show myocardial scar (panel D). There was LV septal wall perfusion abnormality extending from the base to the apex on CMR stress short axis slices (panel E) that was minimally present at rest (panel F). SPECT images also illustrated ischemia in the same region (panel G, stress; panel H, rest). Cardiac catheterization showed total occlusion of the septal branch of the LAD with collaterals from the RCA to the septal branch (panels I and J).

39 patients (29 males) aged 33-78 (mean 54.3 ± 12.4 years) underwent a single treadmill stress study combined with CMR and single photon emission computed tomography (SPECT) imaging. After rest Tc99m SPECT imaging and resting CMR cine imaging, Bruce protocol stress was performed using a fully MRI-compatible treadmill placed adjacent to the scanner table. 12-lead ECG monitoring was performed throughout. At peak stress, Tc99m was injected and allowed to circulate for at least 90 seconds. The patient then quickly returned to the prior position in the magnet for post-exercise cine and perfusion imaging. The patient was then brought out of the magnet for recovery monitoring. After adequate recovery, the patient was sent back into the magnet for recovery cine and resting perfusion imaging, followed by late gadolinium enhancement imaging. After CMR, each patient returned to the adjacent nuclear lab to complete stress SPECT imaging. Images from each modality were reviewed by 3 reviewers blinded to the results of the other modality (Figure 1). Coronary angiography or gated coronary computed tomography was performed in a subset of patients. Luminal stenosis ≥70% was considered to be significant coronary artery disease (CAD).

## Results

Exercise time averaged 10.0±2.9 minutes. Exercise ECG was interpretable during all stages of exercise in 95% of the patients. Stress cine imaging commenced on average at 28±5sec following end of exercise, and stress function and perfusion were completed by 64±7sec, vs. previously reported 42±5sec and 88±8sec, respectively, using a partially modified treadmill in the corner of the scanner room. Agreement between SPECT and CMR for the detection of ischemia and scar was moderate (κ = 0.56). Accuracy for detection of significant CAD in the 21 patients who had coronary angiography was 21/21 for CMR and 17/21 for SPECT (p = 0.13). Follow up at 8-12 months indicated excellent prognosis, with all patients with negative CMR or SPECT having no coronary events.

## Conclusions

Exercise stress CMR using an MRI-compatible treadmill to assess wall motion and perfusion was feasible, accurate, and had good prognostic value in patients with suspected ischemic heart disease. Larger-scale, multicenter studies are needed to confirm our initial experience that suggests comparable accuracy of exercise CMR vs. SPECT to identify myocardial ischemia and infarct scar due to CAD.

## Funding

National Institute of Health (NIH).

